# Fatigue and Quality of Life in Children with Chronic Kidney Disease

**DOI:** 10.3390/children9091414

**Published:** 2022-09-18

**Authors:** Vasiliki Karava, Sofia Goutou, John Dotis, Antonia Kondou, Evangelia Charela, Olympia Dadoudi, Theodoros Eleftheriadis, Ioannis Stefanidis, Nikoleta Printza

**Affiliations:** 1 Pediatric Nephrology Unit, 1st Department of Pediatrics, Hippokratio General Hospital, Aristotle University of Thessaloniki, 54642 Thessaloniki, Greece; 2 BSc Psychology, University of Birmingham, Birmingham B15 2TT, UK; 3 Division of Nephrology, University Hospital of Larissa, University of Thessaly School of Medicine, 41334 Larissa, Greece

**Keywords:** PedsQL, questionnaire, kidney transplantation, dialysis, sleep, school, physical functioning, emotional impact, parent, child

## Abstract

Background: This study investigates the effect of chronic kidney disease (CKD) stage on fatigue and health-related quality of life (HRQoL) in the pediatric population. Material and Methods: The PedsQL (Pediatric Quality of Life Inventory) Multidimensional Fatigue Scale (subcategories: general, sleep/rest, and cognitive fatigue) and HRQoL Generic Core Scales (subcategories: physical, emotional, social, and school functioning) questionnaires were completed by 30 patients aged from 7 to 18 years old with CKD stage 2–4, CKD stage 5 on dialysis (CKD 5D), and kidney transplantation (KTx), as well as their parents. Results: Both low “Total Fatigue” and “Total HRQoL” scores were reported in 16.7% of patients. “Sleep/Rest Fatigue”, “Emotional Functioning”, and “School functioning” were the lowest scored subcategories. CKD 5D/KTx patients presented lower “Sleep/Rest Fatigue” (*p* = 0.022) and, more frequently, low “School Functioning” scores (*p* = 0.029). The “Total HRQoL” score was correlated to the “Total Fatigue” score (rs = 0.625, *p* < 0.001). A low “Sleep/Rest Fatigue” score was associated with low “Physical Functioning”, “School Functioning”, and “Total HRQoL” scores (*p* = 0.016, *p* = 0.001, and *p* = 0.047 respectively). Parents’ HRQoL score was lower than patients’ score on “Physical Functioning” (*p* = 0.040) and “School Functioning” subcategories (*p* = 0.045). Conclusions: Fatigue and disturbed HRQoL are mostly observed in CKD 5D and KTx pediatric patients, and are associated with sleep disorders and school dysfunction. Fatigue affects HRQoL, which is perceived as more deteriorated by the patients’ parents.

## 1. Introduction

Although chronic kidney disease (CKD) is a rare condition in children, its occurrence has increased during the last decades [1]. Apart from the well-known consequences on physical health, recent data suggest that CKD negatively impacts children’s cognitive health and psychosocial status, which may inhibit a successful transition into adulthood [2]. Chronic anemia, metabolic acidosis, uremic toxic accumulation, and cardiovascular disorders that accompany CKD may affect not only the physical, but also the neurocognitive, development of pediatric patients [3]. Specifically, children with CKD may present reduced cognitive function compared to the general population, accompanied by mild deficits across academic skills, executive function, and visual and verbal memory [2]. These deficits may be aggravated by frequent school absenteeism due to hospitalizations and regular hemodialysis sessions, ultimately impairing school functioning [4]. Moreover, the psychological impacts of the disease in a pediatric patient are multiple, including adjustment disorders followed by depression, anxiety, and elimination disorders [5]. Furthermore, behavioral disorders, including emotional problems, hyperactivity, and peer problems, are also reported in CKD pediatric patients [6]. Probable decisive factors for the patient psychological status might involve the CKD duration and stage, but also the family financial status, as well as the parental education level. In clinical practice, multiple health-related quality of life (HRQoL) questionnaires have been applied for the evaluation of HRQoL in pediatric CKD patients, indicating impaired physical, emotional, social, and school functioning [7]. It is, therefore, evident that there is a growing need to investigate and properly manage the risk factors of HRQoL disturbance in order to prevent the occurrence of this condition in this vulnerable population.

Fatigue, defined as the difficulty initiating and sustaining activity and attention tasks, known as physical and mental fatigue, respectively, due to a lack of energy, accompanied by a desire to rest, constitutes a common symptom in chronic diseases, ultimately impairing an individual’s functionality and autonomy [8]. Fatigue has been frequently reported in adult CKD. According to a recent review paper, 20–91% of CKD adult patients suffer from fatigue; severe fatigue is encountered in 5–24% of patients, and its frequency rises in the late stages of the disease [9]. Multiple pathogenetic mechanisms have been implicated in the development of fatigue in the CKD state. Impaired oxygen delivery, attributed to renal anemia, chronic uremia, and cardiovascular comorbidities, contributes to muscular fatigue [9]. Moreover, skeletal muscle deficit, due to metabolic acidosis, chronic inflammation, increased protein catabolism, malnutrition, and mineral bone disorders, induce chronic fatigability in these patients [9]. Neurological complications, especially nerve dysfunctions, mostly observed in patients with CKD stage 5 on chronic dialysis (CKD 5D), may enhance performance fatigability [10]. Other dialysis-related factors, including mode and frequency of dialysis, may also affect the prevalence of this condition in CKD 5D patients [8]. Additionally, sleep disorders, depressive symptoms, anxiety, and stress often coexist in CKD patients with fatigue [8,9]. Fatigue has been associated with adverse clinical outcomes in adult patients, including cardiovascular events, decreased HRQoL, and mortality [8,11]. Conclusively, the prompt diagnosis of this condition is crucial for the management of these patients. Early recognition of fatigue remains a challenge in clinical practice, due to the multidimensional aspect of this symptom, including both physical and cognitive domains. Therefore, the application of multidimensional scale questionnaires seems the most optimal tool for the prompt assessment of chronic fatigue in CKD patients.

In the pediatric CKD population, data regarding fatigue are limited. According to a large-scale cross-sectional study in children with CKD stages 1–4, the “low energy” symptom was observed in 11% of patient reports and in 14% of parent reports; the frequency of this symptom was higher in advanced CKD, and “low energy” was also associated with lower HRQoL scores [12]. The assessment of multidimensional fatigue by employing specific pediatric questionnaires, and the effect of the multidimensional fatigue in HRQoL disturbance have not been investigated yet in the pediatric population. Moreover, data regarding multidimensional fatigue in patients with kidney transplantation (KTx) and with CKD 5D are lacking. The purpose of this cross-sectional study is to investigate the occurrence of multidimensional fatigue, the deterioration of HRQoL, and the interaction between them in pediatric patients with CKD 2–4 stage and with KTx, by using validated pediatric questionnaires. Moreover, our study aims to explore the role of CKD stage on the occurrence of fatigue and HRQoL deficit in this population.

## 2. Materials and Methods

### 2.1. Participants

This cross-sectional study was conducted from March to October 2020 on children and adolescents aged from 7 to 18 years with CKD stage 2–5 or KTx, followed-up in the Pediatric Nephrology Unit of our Department of Pediatrics. Patients with neurological and musculoskeletal disorders not related to CKD, as well as participants unable to complete the questionnaires due to insufficient Greek language knowledge, were excluded. Demographic data, including age, sex, primary kidney disease, and CKD stage, according to the estimated glomerular filtration rate (eGFR) using Schwartz’s formula, were recorded for each patient. The participants were divided into the following three groups based on CKD status: CKD stage 2–4, CKD 5D, and KTx. Moreover, we collected data regarding the parental education level and the highest education level from two parents, or the education level of a single parent in case of a single-parent family. Parent education level was divided into three subcategories: elementary school, high school, and university degree. Finally, we reported the patient residence place (urban, rural).

### 2.2. Procedures

During an outpatient visit, the physician of the pediatric nephrology unit informed the participants about the study protocol, and demanded written consent for study participation. Then, both the patient and parents were asked to fulfill both questionnaires in calm and safe conditions, with a sufficient time provided for completion. The current study analyzed only the fully completed questionnaires. Fatigue and HRQoL were measured using the Greek version of the “PedsQL–Multidimensional Fatigue Scale” and “PedsQoL-Pediatric Quality of Life Inventory” Generic Score Scales questionnaires, respectively.

### 2.3. Instruments

The “PedsQL-Multidimensional Fatigue scale” questionnaire consists of 3 subcategories, including general, sleep/rest, and cognitive fatigue, each of which comprises 6 items. All times are evaluated on a 5-point Likert scale, from 0–4, where 0 = never a problem, 1 = rarely a problem, 2 = sometimes a problem, 3 = often a problem, and 4 = always a problem. For the purposes of our study, the items were reverse-scored and transformed to a 0–100 scale (0 = 100, 1 = 75, 2 = 50, 3 = 25, 4 = 0). The “Total Fatigue” score and the score for each subcategory were measured using the average score of all items and the average score of each subcategory items, respectively. For the purposes of our study, we used the teen report for patients aged from 13 to 18 years old, the child report for patients aged from 8 to 12 years old, and the young child report for patients aged 7 years old. The “PedsQL-Multidimensional Fatigue Scale” has been already used for the evaluation of fatigue in various pediatric populations, including patients with pediatric obesity, and chronic rheumatoid, neuromuscular, and respiratory diseases [13,14,15,16].

The “PedsQL- Pediatric Quality of Life Inventory” Generic Score Scales questionnaire consists of 4 subcategories, including physical, emotional, social, and school functioning, each of which comprises 8, 5, 5, and 5 questions, respectively. As for the “PedsQL-Multidimensional Fatigue scale” questionnaire, we used the teen report for patients aged from 13 to 18 years old, the child report for patients aged from 8 to 12 years old, and the young child report for patients aged 7 years old. The “Total QoL” score and the score for each subcategory were measured using the same procedure that was followed for the fatigue questionnaire. The “PedsQL-Pediatric Quality of Life Inventory” Generic Score Scales questionnaire has been already applied for the evaluation of HRQOL in general [17,18] and various pediatric disease populations, including patients with CKD [12,19,20].

### 2.4. Statistical Analysis

Statistical analysis was conducted using SPSS software (version 25.0). Continuous data were presented as median values and ranges, and categorical data as number and percentages. The comparison of the distribution of on-study parameters between the patient groups based on CKD status were performed using the chi-squared test and the multiple chi-squared test for quantitative independent variables, and Fisher’s exact test and the Kruskal–Wallis test for quantitative independent variables. Spearman’s correlation analysis was applied for the correlation test between fatigue and HRQoL scores. The Wilcoxon signed-rank test was used for the comparison of scores between patients and their parents. Box plot analysis was used for the illustration of patient and parent report scores regarding fatigue and HRQoL questionnaires. A value less than 50 was considered as a low score for total fatigue and HRQoL, as well as for their subcategories. A value of *p* < 0.05 was considered statistically significant.

## 3. Results

In total, 30 children (20 boys, 10 girls) with a median age of 13 (7–18) years old, participated in this study. Of note, two patients were initially excluded from the study because of severe neurological deficits not related to CKD, and two patients were not recruited because of insufficient Greek language knowledge. None of the eligible patients refused to participate in the study. All the questionnaires were fully completed. The patient place of residence was urban in 17 (56.7%) patients and rural in 13 (43.3%) patients. The parent education level involved elementary school in 7 (23.3%), high school in 12 (40%), and a university degree in 11 (36.7%) patients. A single-parent family was observed in only one case.

Among the included patients, 7 patients (5 boys, 2 girls) had undergone KTx, with a median post-transplant time of 7.1 (5.7–8.9) years; 10 patients (6 boys, 4 girls) presented CKD 2–4, consisting of 5 patients with stage 2, 4 with stage 3, and 1 with stage 4; and 13 patients (8 boys, 5 girls) presented CKD 5D, with a median dialysis duration of 4.3 (1.5–6.2) years. Among CKD 5D patients, 10 were on peritoneal dialysis and 3 were on hemodialysis. Primary kidney disease was congenital abnormalities of the kidney and urinary tract in 16 patients, hemolytic uremic syndrome in 5, ciliopathies in 4, focal segmental glomerulosclerosis (FSGS) in 2, polycystic kidney disease in 2, and congenital abnormalities of uric acid metabolism in 1 patient, respectively. In total, confirmed inherited kidney disease was present in 23 (76.7%) patients. The distribution of patient age, sex, frequency of inherited kidney disease, residence place, and parental education level did not significantly differ among the three patient groups (*p* = 0.308, *p* = 0.877, *p* = 0.312, *p* = 0.520, and *p* = 0.212, respectively) (Table 1).

**Figure 1:**
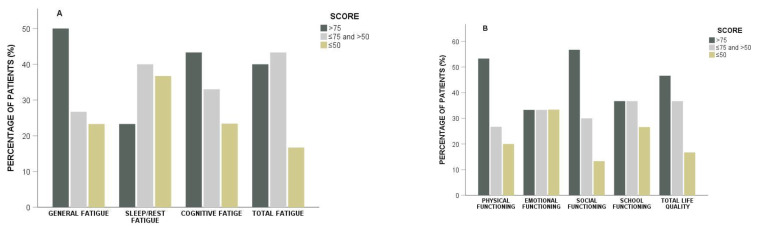
Percentage of patients with scores > 75, ≤75; and >50, ≤50 on (**A**) fatigue and (**B**) health-related quality of life (HRQoL), and their subcategories.

The median scores on “Total Fatigue” and the subcategories, “General Fatigue”, “Sleep/Rest Fatigue”, and “Cognitive Fatigue”, were 68.1 (range 36.1–93.1), 77.1 (45.8–100), 66.7 (29.2–100), 75 (25–100), and 68.1 (36.1–93.1), respectively. The patients’ scores on the subcategory, “Sleep/Rest Fatigue”, were lower compared to that of the other subcategories (*p* = 0.043). Specifically, a score ≤50 was observed for “General Fatigue” in 7 (23.3%) patients, for “Sleep/Rest Fatigue” in 11 (36.7%) patients, for “Cognitive Fatigue” in 7 (23.3%) patients, and for “Total Fatigue” in 5 (16.7%) patients (Figure 1).

**Figure 2:**
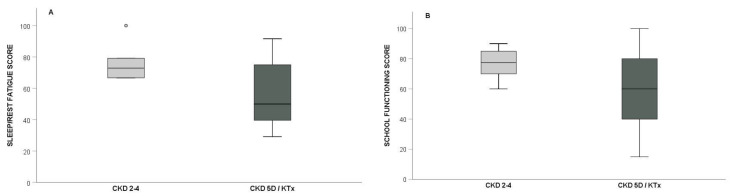
Box plot of (**A**) “Sleep/Rest Fatigue” and (**B**) “School Fucntioning” scores in patients with chronic kidney disease stage 2–4 (CKD 2–4) compared to patients with chronic kidney disease stage 5 on dialysis (CKD 5D) or kidney transplantation (KTx).

The scores regarding fatigue in patients with CKD 2–4, CKD 5D, and KTx are presented in detail in Table 1. Low “General Fatigue” scores were more frequently reported in CKD 5D patients (46.2%) compared to CKD 2–4 (10%) and KTx (0%) patients (*p* = 0.032). Moreover, the prevalence of low “Sleep/Rest Fatigue” scores was significantly higher in both CKD 5D (53.8%) and KTx patients (57.1%) compared to CKD 2–4 patients (0%) (*p* = 0.013). In total, “Sleep/Rest Fatigue” scores were significantly lower in patients under kidney replacement therapy (CKD 5D or KTx) compared to those with CKD 2–4 (*p* = 0.022) (Figure 2). Specifically, low “Sleep/Rest Fatigue” scores were observed in 55% of patients with CKD 5D or KTx, and in no patient with CKD 2–4 (*p* = 0.004). The prevalence of low “Cognitive Fatigue” and “Total Fatigue” scores was higher in CKD 5D and in KTx patients compared to CKD 2–4 patients, but the statistical analysis did not reach significance (*p* = 0.289 and *p* = 0.143, respectively).

The median scores of “Total HRQoL” and the subcategories, “Physical Functioning”, “Emotional Functioning”, “Social Functioning”, and “School Functioning”, were 72.8 (range 35.9–93.5), 78.1 (28.1–100), 67.5 (20–100), 80 (20–100), and 70 (15–100), respectively. Patient scores on the subcategories, “Emotional Functioning” and “School Functioning”, were lower compared to the other subcategories, but the statistical analysis did not reach significance (*p* = 0.246). Specifically, a score ≤ 50 was observed for “Physical Functioning” in six (20%) patients, for “Emotional Functioning” in ten (33.4%) patients, for “Social Functioning” in four (13.3%) patients, for “School Functioning” in eight (26.6%) patients, and for “Total HRQoL” in five (16.7%) patients (Figure 1).

The scores regarding HRQoL in children with CKD 2–4, CKD 5D, and KTx are presented in Table 1. Although low “Physical Functioning” and “Social Functioning” scores were more frequently observed in CKD 5D patients, compared to KTx and CKD 2–4 patients, the statistical analysis did not reach significance (*p* = 0.425 and *p* = 0.325, respectively). The prevalence of “School Functioning” scores tended to be higher among CKD 5D (38.5%) and KTx (42.9%), compared to CKD 2–4 (0%) patients (*p* = 0.062). In total, “School Functioning” scores were lower, whereas the percentage of patients who scored ≤50 on the “School Functioning” subcategory was significantly higher among patients under kidney replacement therapy (CKD 5D or KTx) (*p* = 0.082 and *p* = 0.029, respectively) (Figure 2). Finally, the prevalence of low “Emotional Functioning” and “Total HQRoL” scores did not differ among the three groups (*p* = 0.538 and *p* = 0.693, respectively).

**Figure 3:**
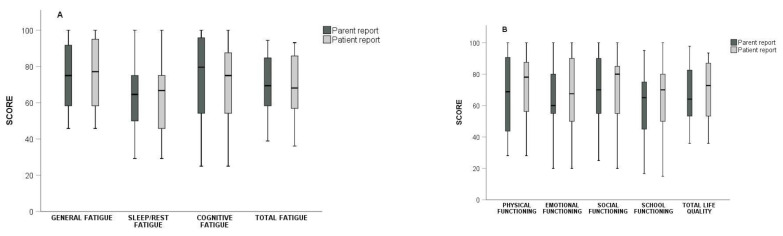
Box plot of patients’ (light gray) and parents’ (dark gray) report scores on (**A**) fatigue and (**B**) health-related quality of life (HRQoL), and their subcategories.

The parental questionnaire was completed by the mother in 22 (73.3%) cases, and by the father in the remaining 8 (26.7%) cases. The parents’ score for “General Fatigue” (*p* = 0.442), “Sleep/Rest Fatigue” (*p* = 0.640), “Cognitive Fatigue” (*p* = 0.414), and “Total Fatigue” (*p* = 0.955) did not differ from those of the patients (Figure 3). On the contrary, parents’ scores were significantly lower for “Total HRQoL” (*p* = 0.029) and for the subcategories, “Physical Functioning” (*p* = 0.040) and “School Functioning” (*p* = 0.045). The scores on the subcategories, “Emotional Functioning” and “Social Functioning”, did not differ between patients and their parents (*p* = 0.530, *p* = 0.154, respectively).

The “Total HRQoL” score was correlated to the “Total Fatigue” score (rs = 0.625, *p* < 0.001), and significant correlations were observed between the fatigue and QoL subcategories’ scores (Table 2). Moreover, significant associations were observed between the fatigue and HRQoL subcategories’ low scores (Table 3). In detail, a low “General fatigue” score was associated with low “Social Functioning” and “Total HRQoL” scores (*p* = 0.031 and *p* = 0.006, respectively); a low “Sleep/Rest Fatigue” score was associated with low “Physical Functioning”, “School Functioning”, and “Total HRQoL” scores (*p* = 0.016, *p* = 0.001 and *p* = 0.047, respectively); a low “Cognitive Fatigue” score was associated with a low “School Functioning” score (*p* < 0.001); and a low “Total Fatigue” score was associated with low “Physical Functioning”, “Emotional Functioning”, “School Functioning”, and “Total HRQoL” scores (*p* = 0.003, *p* = 0.031, *p* = 0.011 and *p* = 0.001, respectively). In total, 24 patients presented a score > 50 for “Total HRQoL” and “Total Fatigue”, four patients presented a score ≤ 50 for “Total HRQoL” and “Total Fatigue”, and two patients presented a score of ≤50 only in “Total HRQoL” or “Total Fatigue”.

## 4. Discussion

Fatigue, both physical and cognitive, is a frequent symptom of chronic diseases. Fatigue is well-described in adult CKD, as a result of physical and mental disorders that accompany the disease [8,9,10]. Moreover, fatigue constitutes a well-recognized feature of frailty syndrome, which is defined as a decline in resistance to minor stress events due to reduced biological reserves, resulting in increased vulnerability to falls, fractures, hospitalization, and mortality [21]. Frailty syndrome is commonly observed in adult CKD patients, with an incidence exceeding 40% in CKD 5D [22]. Interestingly, this condition has been recently reported in the pediatric CKD population, with a higher frequency in advanced stages, and has been associated with a higher hospitalization rate and lower bone mineral density [23,24]. Therefore, although the literature data are limited, the need arises for the systematic recording of fatigue in CKD pediatric patients.

In our study, we used the “PedsQL-Multidimensional Fatigue scale” for the assessment of fatigue. To our knowledge, this is the first study which investigated the prevalence of multidimensional fatigue in CKD pediatric patients. According to our results, a low “Total Fatigue” score was observed in 16.7% of patients. As expected, “General Fatigue” was more prevalent in CKD 5D patients, observed in 46.2% of patients, confirming the major role of CKD chronicity and severity in the manifestation of this symptom. Our findings are in accordance with Roumelioti ME et al.’s study, where children with measured GFR 40- < 50, 30- < 40, or <30 presented 2.07, 2.35, and 2.59 higher odds of experiencing the “low energy” symptom than children with GFR ≥ 50 [12]. The “General Fatigue” symptom is probably the consequence of multiple CKD-related factors, involving chronic uremia, renal anemia, muscle and protein energy wasting, bone mineral disorders, and cardiovascular disease, which are highly encountered in CKD 5D patients [8,9,10]. Moreover, we remarked that “General Fatigue” symptom prevalence was not observed in KTx patients, highlighting the need for the implementation of strategies aimed at reducing the dialysis vintage in the pediatric population. KTx has proven beneficial in terms of mortality and cardiovascular morbidity in the pediatric population [25]. The impact of pre-emptive KTx, defined as KTx before the need for extra-renal epuration therapy, on the prevention of the occurrence of “General Fatigue” in these patients needs further investigation.

Sleep disorders have already been reported in the pediatric CKD population, with a higher prevalence in the late stages [26,27,28]. In a recent meta-analysis, pediatric patients presented a 3.9-fold and a 9.6-fold increased risk of restless legs syndrome and excessive daytime sleepiness compared with controls [28]. Moreover, sleep-disordered breathing and obstructive sleep apnea have been frequently observed in this population, with a prevalence of 22% and 34%, respectively [28]. Although the pathogenesis of this condition has not been thoroughly investigated, multiple factors are linked with its occurrence, including chronic uremia, metabolic acidosis, iron deficiency, and hypertension [26,27]. In the current study, “Sleep/Rest Fatigue” was the major fatigue characteristic, noted in 36.7% of patients, followed by “General Fatigue” and “Cognitive Fatigue”, encountered in 23.3% and 23.3% of patients, respectively. According to the data of the “Sleep/Rest Fatigue” questionnaire items, insomnia, morning tiredness, and sleepiness were the most common sleep problems. It is worth mentioning that low “Sleep/Rest Fatigue” scores were significantly observed in both CKD 5D and KTx patients, affecting 55% of these patients. Sleep disorders have been observed in the adult KTx population, and the regular administration of immunosuppressive drugs, obesity, cardiovascular disease, and allograft loss anxiety have been implicated in its pathogenesis [29]. Similar data are limited in pediatric patients. According to a recent study, sleep-related breathing disorders were remarked in 26% of pediatric kidney-transplant recipients, and were associated with cardiometabolic risk factors, including altered lipid profiles and left ventricular hypertrophy [30]. Conclusively, our results highlight that attention should be paid for the evaluation of sleep disturbances in both CKD 5D and KTx patients, and further studies are required to explore the triggering factors in both patient groups.

In contrast to fatigue, HRQoL has been intensively evaluated in pediatric CKD, with conflicting results among the reported studies. According to some authors, HRQoL scores were lower in CKD, principally in CKD 5D patients, compared to the healthy population [6,31,32,33], whereas in other studies, HRQoL was not significantly deteriorated in CKD pediatric patients [34,35]. Moreover, whereas in some studies, KTx was associated with improved physical status and educational activity, a higher degree of self-esteem, and an enhanced development of social skills compared to CKD 5D patients [31,32], HRQoL did not differ between KTx and CKD 5D patients in other studies [33,36]. Furthermore, according to the literature data, patients on peritoneal dialysis presented a better HRQoL compared to those on hemodialysis, possibly due to the higher level of school and extracurricular activity attendance [19,37].

In our study, a low “Total HRQoL” score was observed in 16.7% of pediatric CKD patients, whereas low “Emotional functioning” and “School functioning” scores were the most frequently observed characteristics of disturbed HRQoL. We also remarked that patients with CKD 5D or KTx presented significantly lower “School Functioning” scores compared to CKD 2–4 patients. In detail, low “School Functioning” scores were observed in 40% of patients with CKD 5D or KTx. According to the data of “School Functioning” questionnaire items, these patients commonly presented attenuated classroom attention and working memory, ultimately burdening their school performance. School absences due to regular consultations or dialysis sessions may also contribute to the learning disabilities. Supplementary in-class and out-class educational activities and the enhancement of the hospital quality of educational activity during dialysis sessions might improve the learning difficulties in this vulnerable population.

The interaction between multidimensional fatigue and HRQoL has not been previously investigated in the pediatric CKD population. In the current study, the “Total Fatigue” score was correlated to the “Total HRQoL” score, and significant correlations were also remarked between the fatigue and HRQoL subcategories’ scores. Moreover, a low “Sleep/Rest Fatigue” score, which was, as previously reported, the most frequent fatigue characteristic, was significantly associated with low “Physical Functioning”, “School Functioning”, and “Total HRQoL” scores. In general, adequate sleep contributes to physical and mental child health, whereas sleep disorders may have a devastating effect on a child’s behavior, learning ability, and physical development [26,27]. Our results highlight the deleterious effect of fatigue on HRQoL in pediatric CKD, and indicate the need for the systematic screening of multidimensional fatigue.

Prompt medical interventions in cases of fatigue are probably the key in order to ameliorate patient HRQoL and well-being. In adult studies, physical activity, erythropoiesis-stimulating agents, bicarbonate supplementation, and vitamin D administration have been proven beneficial for attenuating fatigue occurrence in CKD 5D patients [9]. Similar studies are lacking in the pediatric population. Nevertheless, given that physical fatigue is primarily affected by bone and muscle status, the protection of musculoskeletal health seems crucial for preventing fatigue in this population [23]. The management of cognitive and sleep/rest fatigue remains a challenge in clinical practice. Collaboration between schoolteachers and the patient’s physician may be helpful to overcome the school difficulties experienced in patients with cognitive fatigue, whereas special educational services based on each patient need may be necessary in cases of severe cognitive fatigue. Moreover, there is growing evidence that melatonin production is decreased and circadian rhythms of melatonin are altered with the progression of CKD [38]. The possible benefits of melatonin therapy in CKD-related sleep disorders are currently under question.

In the current study, parents’ fatigue scores did not differ from that of patients, emphasizing that this condition is perceived to the same degree by both patients and their relatives. Nevertheless, parents’ scores were significantly lower compared to those of patients for “Total HRQoL”, “Physical functioning”, and “School functioning”. Similarly, more favorable patient reports, compared to parent reports, on HRQoL have been observed in other pediatric CKD studies [22,23]. These differences may indicate problematic communication patterns between family members, and may reflect a deteriorated HRQoL of the parents themselves [19,23]. In other words, CKD may affect parents’ HRQoL more than that of the patients. The demands of CKD, as a chronic illness, are multiple and may result in extreme parenting behaviors, such as abusive and over-involved parenting. The psychological support of both patients and their relatives seems necessary to overcome such issues.

Our study has several limitations. Firstly, the limited number of included patients, with a variety of ages, precludes us from making firm conclusions. Moreover, the cross-sectional results of our study need to be confirmed in large longitudinal analyses. Furthermore, our study concerned only Greek pediatric patients; therefore, our results might not be representative of the European pediatric CKD population. Additionally, other factors that may affect QoL, such as patient comorbidities and parent socio-economic status, have not been taken into account in our study.

A strength of our study is that we investigated both multidimensional fatigue and HRQoL in our cohort, allowing us to explore the interaction between the two conditions in CKD pediatric patients. Moreover, we included patients with both CKD 2–5D and KTx, which permitted us to compare the prevalence of multidimensional fatigue and altered HRQoL across the full spectrum of CKD. Furthermore, this is the first study which employed a multidimensional fatigue questionnaire validated for pediatric use in CKD pediatric patients, allowing us to explore all domains of fatigue in this vulnerable population.

Conclusively, according to our results, general fatigue is observed in children with CKD, with a higher frequency of occurrence in CKD 5D. Sleep disorders represent the main fatigue characteristic, encountered in both CKD 5D and KTx patients. HRQoL deterioration is associated with emotional disorders and learning difficulties, especially in CKD 5D and KTx patients. Fatigue seems to affect the patient HRQoL. Finally, although fatigue is perceived equally by children and their parents, the parents’ perception of their children’s HRQoL is worse than that of the children themselves.

## Figures and Tables

**Table 1:** Distribution of scores regarding fatigue and Health Related- Quality of Life (HRQoL) and their subcategories in pediatric patients with chronic kidney disease (CKD) stage 2–4, CKD stage 5 on chronic dialysis (CKD 5D), and kidney transplantation (KTx). Continuous data are presented as median values (ranges), whereas categorical data are presented as numbers (percentages).

	CKD 2–4 10 Patients	CKD 5D 13 Patients	KTx 7 Patients	*p*
Demographic data				
Age	11.5 (7–18)	13 (7–18)	14 (8–18)	0.308
Sex (male)	6 (60%)	8 (61.5%)	5 (71.4%)	0.877
Inherited kidney disease	6 (60%)	11 (84.6%)	6 (85.7%)	0.312
Residence place (urban)	7 (70%)	6 (46.1%)	4 (57.1%)	0.520
Parental education level				0.212
Elementary school	1 (10%)	5 (38.4%)	1 (14.2%)	
High school	3 (30%)	6 (46.2%)	3 (42.9%)	
University Degree	6 (60%)	2 (15.4%)	3 (42.9%)	
Fatigue scores				
General Fatigue	89.6 (45.8–100)	62.5 (45.8–100)	83.3 (58.3–100)	0.076
Sleep/Rest Fatigue	72.9 (66.6–100)	50 (29.2–79.2)	50 (33.3–91.6)	0.071
Cognitive Fatigue	83.4 (41.7–100)	66.7 (25–87.5)	58.3 (50–100)	0.215
Total Fatigue	84.7 (55.5–93.1)	59.7 (36.1–85.8)	69.4 (47.2–93)	0.052
Low Fatigue scores				
General Fatigue	1 (10%)	6 (46.2%)	0 (0%)	0.032
Sleep/Rest Fatigue	0 (0%)	7 (53.8%)	4 (57.1%)	0.013
Cognitive Fatigue	1 (10%)	3 (23.1%)	3 (42.9%)	0.289
Total Fatigue	0 (0%)	4 (30.8%)	1 (14.3%)	0.143
HRQoL scores				
Physical Functioning	87.5 (43.75–100)	75 (28.1–100)	78.1 (40.6–87.5)	0.210
Emotional Functioning	77.5 (30–100)	55 (20–95)	65 (25–95)	0.324
Social Functioning	82.5 (50–100)	70 (35–100)	70 (55–85)	0.173
School Functioning	77.5 (60–90)	60 (17–100)	60 (15–90)	0.209
Total HRQoL	87 (48.9–91.7)	67.4 (35.9–93.5)	71.7 (45.7–80.4)	0.126
Low HRQoL scores				
Physical Functioning	1 (10%)	4 (30.8%)	1 (14.3%)	0.425
Emotional Functioning	2 (20%)	5 (38.5%)	3 (42.9%)	0.538
Social Functioning	1 (10%)	3 (23.1%)	0 (0%)	0.326
School Functioning	0 (0%)	5 (38.5%)	3 (42.9%)	0.064
Total HRQoL	1 (10%)	3 (23.1%)	1 (14.3%)	0.693

**Table 2:** Correlation between fatigue and health-related quality of life (HRQoL) scores.

	Physical Functioning	Emotional Functioning	Social Functioning	School Functioning	Total HRQoL
General Fatigue	rs = 0.434, *p* = 0.017	rs = 0.409, *p* = 0.025	rs = 0.319, *p* = 0.086	rs = 0.417, *p* = 0.019	rs = 0.504, *p* = 0.004
Sleep/Rest Fatigue	rs = 0.481, *p* = 0.007	rs = 0.302, *p* = 0.105	rs = 0.459, *p* = 0.011	rs = 0.458, *p* = 0.011	rs = 0.503, *p* = 0.005
Cognitive Fatigue	rs = 0.565, *p* = 0.001	rs = 0.455, *p* = 0.011	rs = 0.513, *p* = 0.004	rs = 0.564, *p* = 0.001	rs = 0.654, *p* < 0.001
Total Fatigue	rs = 0.564, *p* = 0.001	rs = 0.414, *p* = 0.023	rs = 0.498, *p* = 0.005	rs = 0.548, *p* = 0.002	rs = 0.625, *p* < 0.001

**Table 3:** Association between low fatigue (score ≤50) and health-related quality of life (HRQoL) scores.

	Physical Functioning			Emotional Functioning			Social Functioning			School Functioning			Total HRQoL		
	>50 24 pts	≤50 6 pts	*p*	>50 20 pts	≤50 10 pts	*p*	>50 26 pts	≤50 4 pts	*p*	>50 22 pts	≤50 8 pts	*p*	>50 25 pts	≤50 5 pts	*p*
General Fatigue	4 (16.7%)	3 (50%)	0.120	3 (15%)	4 (40%)	0.181	4 (15.4%)	3 (75%)	0.031	4 (18.2%)	3 (37.5%)	0.345	3 (12%)	4 (80%)	0.006
Sleep/ Rest Fatigue	6 (25%)	5 (83.3%)	0.016	5 (25%)	6 (60%)	0.108	9 (34.6%)	2 (50%)	0.611	4 (18.2%)	7 (87.5%)	0.001	7 (28%)	4 (80%)	0.047
Cognitive Fatigue	4 (16.7%)	3 (50%)	0.120	3 (15%)	4 (40%)	0.181	6 (23.1%)	1 (25%)	1	1 (4.5%)	6 (75%)	<0.001	4 (16%)	3 (60%)	0.068
Total Fatigue	1 (4.2%)	4 (66.7%)	0.003	1 (5%)	4 (40%)	0.031	3 (11.5%)	2 (50%)	0.119	1 (4.5%)	4 (50%)	0.011	1 (4%)	4 (80%)	0.001

## Data Availability

The original contributions presented in the study are included in the article; further inquiries can be directed to the corresponding author.
